# Relationships Between Nurses' Self‐Leadership Practices, Professional Autonomy, Job Satisfaction and Intention to Leave: A Structural Equation Modelling Approach

**DOI:** 10.1111/jan.70316

**Published:** 2025-10-23

**Authors:** Katja Pursio, Anu Nurmeksela, Santtu Mikkonen, Tarja Kvist

**Affiliations:** ^1^ Department of Nursing Science, Faculty of Health Sciences University of Eastern Finland Kuopio Finland; ^2^ Department of Environmental and Biological Sciences University of Eastern Finland Kuopio Finland; ^3^ Department of Technical Physics University of Eastern Finland Kuopio Finland; ^4^ Wellbeing Services County of Central Finland Jyväskylä Finland

**Keywords:** intention to leave, job satisfaction, professional autonomy, registered nurse, self‐leadership, structural equation modelling

## Abstract

**Aim:**

To explain the relationships between nurses' self‐leadership and professional autonomy, job satisfaction, and intention to leave the profession.

**Design:**

A descriptive cross‐sectional study design.

**Methods:**

A total of 230 registered nurses responded to a survey including a Finnish version of the Dempster Practice Behaviour Scale and the Revised Self‐Leadership Questionnaire in fall 2024. Structural equation modelling was used to test hypotheses.

**Results:**

Nurses assessed their self‐leadership practices as moderately good. The model indicated that self‐goal setting, evaluating beliefs and assumptions, and job satisfaction have positive relationships with professional autonomy, while self‐reward and self‐punishment have negative relationships with it. It also demonstrated that natural reward strategies have a positive relationship with job satisfaction, while self‐punishment has a negative relationship with it. Nurses' professional autonomy and job satisfaction reduce their intention to leave, while evaluating beliefs and assumptions increase it.

**Conclusion:**

Goal setting and using constructive mindsets develop ways of thinking that positively impact nurses' autonomy. This, in turn, leads to higher job satisfaction and lower intention to leave. Natural reward strategies that involve performing meaningful tasks surrounded by empowering people increase job satisfaction. However, not all self‐leadership strategies are beneficial: self‐punishment can lower professional autonomy and job satisfaction. Additionally, evaluating one's own beliefs and assumptions might increase the intention to leave due to reflective thoughts about the profession.

**Implications:**

Implementing professional autonomy and self‐leadership practices in organisational structures enhances nurses' valuable role. Empowering leadership encourages nurses to set goals, evaluate beliefs and assumptions, and reward themselves. Moreover, nurses' self‐punishment can be avoided with a healthy, open work environment. Self‐leadership skills should be strengthened in nursing education to prepare nurses for work demands.

**Reporting Method:**

The STROBE checklist.

**Patient or Public Contribution:**

No patient or public contribution.

**Summary**

- What problem did the study address?
  ○ Nurses' self‐leadership is still understudied, and more research is needed globally. Self‐leadership and professional autonomy are key prerequisites for nurses to influence their own work and job satisfaction, so their relationships need to be explored in more detail to increase nurses' engagement and retention.
- What were the main findings?
  ○ Nurses' self‐goal setting, evaluating beliefs and assumptions, job satisfaction, self‐reward, and self‐punishment have relationships with professional autonomy. Natural reward strategies and self‐punishment have relationships with job satisfaction. Nurses' professional autonomy, job satisfaction, and evaluating beliefs and assumptions are associated with the intention to leave.
- Where and on whom will the research have an impact?
  ○ Promoting nurses' professional autonomy and self‐leadership practices could be a crucial step in sustaining the nursing workforce. Study findings will benefit clinical nurses, nursing leaders, healthcare organisations, and educational institutions.

## 1 Introduction

A calling to nursing is no longer enough to attract and retain nurses. Nurses increasingly display turnover intentions, or at worst, the intention to leave the entire profession if daily work does not produce job satisfaction. Europe is one of three regions, together with the Americas and the Western Pacific, with the highest density of nurses (76.9 nurses/10,000 population). A total of 78% of all nurses work in these countries, which represent just under half of the world's population. From a global perspective, it is particularly important that these regions strive to improve the retention of existing nursing staff by improving working conditions and job satisfaction, instead of increasing international recruitment from regions where nursing shortages are even more critical (WHO 2025).

Healthy work environments and nursing leadership are key factors in increasing nurses' job satisfaction (Wong 2024), but attention has also been paid to nurses' professional autonomy and opportunities to influence their own work (Bartmess et al. 2024; Pursio, Kankkunen, and Kvist 2024). The way each nurse acts is individual, and the need for autonomy varies between nurses, forcing leaders to consider how organisational structures can enhance and support nurses' full potential, sense of meaning, and enjoyment at work. Equally, nurses themselves ought to find ways to make their hectic and sometimes very stressful work rewarding. They need to bear responsibility for sustainable working practices and their own job satisfaction. Self‐leadership as a comprehensive process of self‐influence at work (Knotts et al. 2021) is an important resource for both nurses and health care organisations, and it is essential to better understand its role in nursing.

## 2 Background

Nurses experience work engagement when they are involved physically, mentally, and emotionally with work (Al Mamari and Groves 2023). Specifically, engagement is high when nurses feel valued in their profession. Several organisational factors, such as work culture, leadership, teamwork, resources, and autonomy affect the state of engagement (Aungsuroch et al. 2024). Therefore, nurses' engagement can be strengthened externally with flexible work, open‐door policies, feedback, and rewards (Al Mamari and Groves 2023), as well as by involving them in decision making (Baghdadi et al. 2020).

Autonomy and organisational support enhance nurses' work engagement; thus, nurses experience control and responsibility at work (Junttila et al. 2023; Kohnen et al. 2023). When nurses have professional autonomy, they are more likely to work with intrinsic motivation, energy, and enthusiasm to achieve goals. This may reflect beneficial organisational citizenship behaviour and increase optimism and engagement in nurses. Moreover, some individual characteristics such as self‐efficacy, resilience, and optimism have also been found to foster nurses' work engagement (Chen et al. 2024). Specifically, nurses' self‐efficacy can enhance their ability to utilise strengths at work and reduce turnover intention (Chu et al. 2021).

Previous studies show that similar factors affect not only engagement but also nurses' job satisfaction. The nursing practice environment, including control over nursing practice, is one of the most significant factors influencing nurses' job satisfaction (Kagan et al. 2021; Pursio, Kankkunen, Mikkonen, and Kvist 2024). Autonomy, as a basic psychological need, is essential to intrinsic motivation and satisfaction, which manifests itself in well‐being (Ryan and Deci 2017). Previous studies highlight the relationship between nurses' professional autonomy and job satisfaction (Labrague et al. 2019; Pursio, Kankkunen, Mikkonen, and Kvist 2024), as well as the negative relationship between nurses' autonomous motivation and their intention to leave the profession (Wei et al. 2023; Gagnon et al. 2024).

Nurses' professional autonomy comprises independence in decisions and actions, along with the ability to influence working conditions (Bartmess et al. 2022). In addition to job satisfaction and engagement, it has been found to be positively related to patient safety activities (Yuk and Yu 2023). Nurses who experience more autonomy at work suffer less emotional exhaustion (Lee and Chang 2022) and fewer job demands, such as role ambiguity and time pressure (Ruotsalainen et al. 2023).

Still, professional autonomy is useless if individuals do not know how to utilise it. Employees with self‐leadership skills strive for optimal performance in accordance with the goals they set for themselves and take responsibility for their actions (van Dorssen‐Boog et al. 2022). Self‐leadership is often described as a multidimensional process in which an individual strives to control their own behaviour through naturally rewarding actions and constructive thinking patterns. It is a comprehensive self‐influence process, whereby it is possible to choose an internally motivating way to perform (Neck et al. 2019). In self‐leadership, the goal is, aligning with conventional leadership, to identify proactive influence strategies that can change behaviour, attitudes, and results (Knotts et al. 2021). One important aspect of self‐leadership is that it enables ownership of an individual's experience through the actions they perform. Thus, regardless of management or organisational constraints, it is still possible for the individual to take ownership of the work and influence issues within their control (Neck et al. 2019).

Three types of self‐leadership strategies influence personal effectiveness and can be used to improve self‐motivation and self‐direction. First, behaviour‐focused strategies include self‐observation, self‐goal setting, self‐reward, self‐punishment, and self‐cueing. These guide behaviour in the desired direction to achieve outcomes. Second, natural reward strategies increase feelings of joy, self‐control, and competence. These focus resources on the most suitable working methods and finding pleasant aspects of each task. Third, constructive thought pattern strategies include positive self‐talk, evaluating beliefs and assumptions, and visualising successful performance. (Neck et al. 2019) Findings from self‐leadership studies show numerous positive effects on employee resources, attitudes, efficiency, and well‐being. Self‐leadership has positive relationships with self‐efficacy and job performance, as well as with job satisfaction and work engagement. Individuals who exercise self‐leadership are usually conscientious and fill their jobs with naturally motivating tasks to increase their satisfaction. (Knotts et al. 2021).

The healthcare environment has become increasingly demanding due to economic uncertainty, social inequality, and overlapping health crises and emergencies (WHO 2025), necessitating proactive self‐direction and responsibility from nurses. Over the past decade, there has been an increased interest in studying self‐leadership in the nursing context. This is an important trend because nurses usually work with insufficient resources in hectic and stressful environments, balancing the demands of patients, relatives, and management (Neck et al. 2019). A recent scoping review raised four main themes from previous studies on nurses' self‐leadership. First, self‐leadership is seen as a category of internal skills that increases with experience. Second, it improves work performance through self‐efficacy and proper communication competence. Third, it improves work well‐being. Finally, it thrives in favourable work environments characterised by suitable work conditions and supportive management (Pursio et al. 2025).

Nurses' self‐leadership, however, is still an understudied topic, and the existing research focuses mainly on Asian countries such as South Korea (Pursio et al. 2025); thus, more research is needed globally. In addition, self‐leadership and professional autonomy are both key prerequisites for nurses to influence their own work and job satisfaction, so their relationships must be studied in more detail to increase nurses' engagement and retention.

## 3 The Study

The aim of this study is to explain relationships between nurses' self‐leadership and professional autonomy, job satisfaction, and intention to leave the profession in one Wellbeing Services County in Finland. The hypotheses are as follows:

Hypothesis 1 *Nurses' self‐leadership practices are positively related to their (a) professional autonomy and (b) job satisfaction*.

Hypothesis 2 *Nurses' self‐leadership practices decrease their intention to leave the profession*.

## 4 Methods/Methodology

### 4.1 Study Design

The study used a descriptive cross‐sectional design and was reported according to the STROBE checklist (Table S2).

### 4.2 Study Setting and Sampling

The participants worked as registered nurses (RNs) in one of the 21 Wellbeing Services Counties in Finland. This Wellbeing Services County has one central hospital and several health care centers and outpatient clinics. Approximately 2300 RNs from different clinical settings, with contracts of at least one month, were invited to participate in the study. Midwives, as they have dual degrees and are registered nurses in Finland, were included if their job descriptions corresponded to the duties of the RNs. Nurses with managerial, education, or specialist positions were excluded.

Sample size was determined by power calculation with a predefined 80% confidence level and a 5% margin of error, yielding a recommended sample size of 154 participants (Lakens 2022). The number of nurses participating in the study (*n* = 230) exceeded the recommended sample size.

### 4.3 Data Collection

The data were collected online from October to November 2024. The contact person forwarded the research bulletin and the electronic survey to the RNs through nurse directors and managers. Reminders were sent during the data collection period.

### 4.4 Instruments

The electronic survey included three sections. Nurses' perceptions of their professional autonomy and self‐leadership were collected using two validated instruments, specifically the Finnish version of the Dempster Practice Behaviour Scale and the Revised Self‐Leadership Questionnaire. In addition, a background information section included questions regarding age, gender, education, and years of nursing experience. Nurses assessed their current job satisfaction on a scale of 0–10 and intention to leave the profession on a 5‐point Likert scale (1: daily; 2: weekly; 3: monthly; 4: sometimes; 5: never) (Sihvola et al. 2023).

#### 4.4.1 Finnish Version of the Dempster Practice Behaviour Scale

Professional autonomy was explored with the Finnish version of the Dempster Practice Behaviour Scale (FI‐DPBS) (Pursio, Kankkunen, and Kvist 2024), which was originally developed to understand and estimate the definitions and dimensions of autonomy in practice (Goolsby et al. 2020). The FI‐DPBS has 24 items, six fewer than the original instrument, categorised into five dimensions of professional autonomy: actualization (5 items related to control, taking action, and determination); valuation (6 items related to satisfaction, respect from oneself and others); authority (6 items related to power, having rights, and legitimacy); empowerment (4 items related to permission and not experiencing constraints); and readiness (3 items related to competence, growth, and responsibility). The items were rated on a 5‐point scale, with a higher number indicating a greater degree of professional autonomy. The content validity index and Cronbach's *α* value of the FI‐DPBS have been reported as 0.94 and 0.89, respectively (Pursio, Kankkunen, and Kvist 2024).

#### 4.4.2 The Revised Self‐Leadership Questionnaire

The Revised Self‐Leadership Questionnaire (RSLQ) measures self‐leadership skills, behaviours, and cognitions. It consists of nine subscales with 35 items, which reflect behaviour‐focused, natural reward, and constructive thought strategies. Five factors (self‐goal setting, self‐reward, self‐punishment, self‐observation, self‐cueing) with 18 items represent behaviour‐focused strategies, one factor with five items represents natural reward strategies, and three factors (visualising successful performance, self‐talk, and evaluating beliefs and assumptions) with 12 items represent constructive thought pattern strategies. Self‐assessed items were scored on a five‐point Likert scale from 1 = Not at all accurate to 5 = Completely accurate. Cronbach's alpha varies between factors from 0.74 to 0.93 (Houghton and Neck 2002).

Based on principal component analysis, the Finnish version of the RSLQ consists of seven components with 31 items (*reference: Authors, accepted*). The structure was validated with confirmatory factor analysis (CFA) with maximum likelihood extraction. As a result, one item was moved from the component of self‐punishment to the component of self‐goal setting, achieving a good model fit (RMSEA 0.050, TLI 0.931, CFI 0.939). Final factors of the Finnish RSLQ are self‐goal setting (7 items), self‐reward (3 items), self‐punishment (4 items), self‐cueing (3 items), natural reward strategies (4 items), visualising successful performance (6 items), and evaluating beliefs and assumptions (4 items). These factors describe self‐leadership practices. The content validity index and the Cronbach's α value of the Finnish RSLQ are 0.96 and 0.93, respectively.

### 4.5 Data Analysis

The background information from the RNs was analyzed using descriptive statistics (percentage, mean, standard deviation). The FI‐DPBS total score, including all items, was analyzed as a mean score, considering that four items were reverse scored (Pursio, Kankkunen, and Kvist 2024). Self‐leadership practices and job satisfaction were analyzed using mean scores. In accordance with the statistical software's assumption that variables must be either linear or dichotomous, the intention to leave the profession was re‐coded into two categories (at least once a month or less than monthly) for further analysis and analyzed as percentages.

Structural equation modelling (SEM) was used to test the study hypotheses. Missing single values (*n* = 21 and *n* = 25 in the FI‐DPBS and RSLQ, respectively) were observed to be missing at random (MAR). SEM proceeded through five model testing steps: model specification, identification, estimation, evaluation, and modification. Maximum likelihood extraction was used. As missingness would decrease the number of responses in use by 20%, missing values were imputed using regression imputation, which estimates missing entries based on linear relationships among observed variables under the assumption of data missing at random. This method preserves the covariance structure of the data and enables the use of all cases in model estimation. The statistical significance of parameter estimates and examination of modification indices (MI) determined if the paths were deleted from or added to the model (Kline 2016). Total, direct, and indirect effects were computed. The model adequacy in SEM was assessed using the root mean square error of approximation (RMSEA), the comparative fit index (CFI), and the Tucker–Lewis fit index (TLI). Model fit was considered acceptable with RMSEA values < 0.1 and CFI and TLI values > 0.9 (Kline 2016). Statistical analyses were performed in SPSS Statistics 29 for Windows and SPSS AMOS 29 Graphics (SPSS Inc., Chicago, IL).

### 4.6 Ethical Considerations

Ethical approval and consent to participate were obtained from the Committee on Research Ethics at the University of Eastern Finland on September 12, 2024 (Statement No 17/2024), while Wellbeing Services County provided study permission on October 20, 2024 (Hyvaks 20.102024). Permission for the use of the instruments was obtained from their copyright holders via e‐mail. RNs received an electronic research information sheet via the contact person and confirmed their voluntary consent before the electronic survey was made available. Responses were anonymous. All research phases were carried out in accordance with the Declaration of Helsinki (World Medical Association 2013).

## 5 Results

### 5.1 Demographic Characteristics, Professional Autonomy, Job Satisfaction, Self‐Leadership Practices, and Intention to Leave

Altogether, 230 registered nurses participated in the study. Ninety‐three percent of them were women, and the most common level of education was a bachelor's degree from a university of applied sciences (63.1%). The nurses' average age was 44.2 years (SD 10.7), and they had an average of 16.1 years' nursing experience (SD 10.4).

Nurses assessed their professional autonomy as 3.74 (SD 0.62) out of 5 and job satisfaction as 7.3 (SD 1.7) out of 10. The mean scores for nurses' self‐leadership practices were 3.42 for self‐goal setting (SD 0.70), 2.47 for self‐reward (SD 0.76), 2.98 for self‐punishment (SD 1.00), 2.81 for self‐cueing (SD 0.95), 3.56 for natural reward strategies (SD 0.76), 2.65 for visualising successful performance (SD 0.94), and 3.39 for evaluating beliefs and assumptions (SD 0.71) (Table 1). In total, 35.7% of the nurses considered leaving the profession at least once a month, while 64.3% considered it less often.

**TABLE 1:** Nurses' professional autonomy, job satisfaction, and self‐leadership practices (*n* = 230).

Measurements	Mean (SD)
Professional autonomy^a^	3.74 (0.62)
Job satisfaction^b^	7.30 (1.7)
Self‐leadership^a^	
Self‐goal setting	3.42 (0.70)
Self‐reward	2.47 (0.76)
Self‐punishment	2.98 (1.00)
Self‐cueing	2.81 (0.95)
Natural reward strategies	3.56 (0.76)
Visualising successful performance	2.65 (0.94)
Evaluating beliefs and assumptions	3.39 (0.71)

^a^ Scale: 1 = lowest, 5 = highest.

^b^ Scale: 1 = lowest, 10 = highest.

### 5.2 Self‐Leadership Practices Related to Nurses' Professional Autonomy, Job Satisfaction, and Intention to Leave the Profession

The model indicated that self‐goal setting (*B* 0.16, *p* < 0.001), evaluating beliefs and assumptions (*B* 0.15, *p* < 0.001), and job satisfaction (*B* 0.10, *p* < 0.001) has positive direct relationships with professional autonomy, while self‐reward (*B* –0.06, *p* = 0.013) and self‐punishment (*B* –0.12, *p* < 0.001) have negative relationships with it. The model also demonstrated that natural reward strategies (*B* 0.44, *p* = 0.002) have direct positive relationships with job satisfaction, while self‐punishment (*B* –0.30, *p* = 0.006) has negative relationships with it. Based on the findings, Hypothesis 1 can be only partially confirmed.

Nurses' professional autonomy (*B* 0.24, *p* < 0.001) and job satisfaction (*B* 0.12, *p* < 0.001) were positively related to their intention to leave; in other words, these factors reduced the intention. On the other hand, evaluating beliefs and assumptions (*B* –0.11, *p* = 0.004) was negatively related to the intention to leave. Thus, Hypothesis 2 was not supported.

**FIGURE 1:**
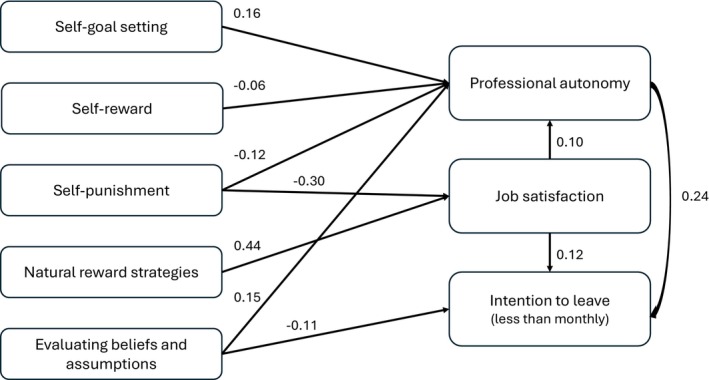
Nurses' self‐leadership practices related to professional autonomy, job satisfaction, and intention to leave (direct effects).

Two self‐leadership practices, namely self‐cueing and visualising successful performance, had no statistically significant relationships with professional autonomy, job satisfaction, or intention to leave and were thus excluded from the model. The presented model fits the imputed data well (RMSEA 0.048, CFI 0.991, TLI 0.971). Figure 1 presents the direct effects and Table 2 presents the standardised total, direct, and indirect effects. Regression weights, both unstandardized and standardised, can be found in File S1.

**TABLE 2:** Standardised total, direct, and indirect effects of self‐leadership practices on nurses' professional autonomy, job satisfaction, and intention to leave profession.

Path	Standardised total effects	Standardised direct effects	Standardised indirect effects
Professional autonomy			
Self‐goal setting	0.244	0.244	0
Self‐reward	−0.147	−0.147	0
Self‐punishment	−0.323	−0.255	−0.068
Natural reward strategies	0.076	0	0.076
Evaluating beliefs and assumptions	0.233	0.233	0
Job satisfaction	0.390	0.390	0
Job satisfaction			
Self‐punishment	−0.175	−0.175	0
Natural reward strategies	0.195	0.195	0
Intention to leave			
Self‐goal setting	0.055	0	0.055
Self‐reward	−0.033	0	−0.033
Self‐punishment	−0.146	0	−0.146
Natural reward strategies	0.098	0	0.098
Evaluating beliefs and assumptions	−0.115	−0.168	0.052
Professional autonomy	0.226	0.226	0
Job satisfaction	0.505	0.417	0.088

*Note:* RMSEA 0.048, CFI 0.991, TLI 0.971, *p* ≤ 0.01 or *p* < 0.001.

## 6 Discussion

The aim of this study was to test whether self‐leadership practices can increase nurses' professional autonomy and job satisfaction, as well as reduce their intention to leave the profession. Investing in nurses' professional growth, job satisfaction, and engagement is above all for the benefit of patients (Wong 2024) and is thus an essential target for attention and development in nursing.

The Finnish version of the RSLQ has two fewer factors (self‐observation and self‐talk) than the original. Self‐leadership theory states that not all self‐leadership behaviours are of equal value in all situations, and they can be emphasised differently by different people (Neck et al. 2019). The registered nurses in this study rated their own self‐leadership practices at a moderately good level because the neutral point is usually placed below the midpoint of the scale due to the positive nature of self‐leadership (Dunaetz et al. 2024). This is an important finding, as previous research has shown that nurses' self‐leadership improves their competence, innovativeness, and job performance (Pursio et al. 2025) and potentially prevents and reduces environmental stress (Neck et al. 2019).

Structural equation modelling showed that nurses who set more personal goals and evaluated more beliefs and assumptions experienced stronger professional autonomy. It would therefore seem that these nurses are more determined in their actions. Furthermore, nurses who reported higher job satisfaction experienced stronger professional autonomy. Professional autonomy is not a privilege given from outside but rather must be taken by the individual. Nurses who are satisfied with their work have the resources to do this. This connection could just as well work the other way around, and there is strong evidence that professional autonomy and job satisfaction are associated with each other (Labrague et al. 2019; Pursio, Kankkunen, Mikkonen, and Kvist 2024; Wong 2024). When nurses feel that they can influence both clinical decision making and, more broadly, their own actions and working conditions, and they are surrounded by favourable nursing environments and leadership, the psychological need for autonomy is fulfilled and satisfaction increases (Ryan and Deci 2017). However, previous studies also present opposite findings. Both Kanninen et al. (2022) and Bartmess et al. (2024) reported that nurses experienced an illusion of autonomy while participating in a nurse staff committee, without a real opportunity to influence. Organisational structures and workplace cultures should be critically assessed to determine if they really support nurses' opportunities to engage in decision making (Bartmess et al. 2022). Nurses' roles are diverse and multifaceted, and sometimes nurses may be the sole providers of healthcare services in specific settings or at specific times of day (WHO 2025). Therefore, their independence—and ability to control their practice—need to be continuously strengthened.

We found that nurses who implemented more self‐punishment experienced lower professional autonomy. Where professional autonomy is based on intrinsic motivation, self‐punishment could be seen as a form of self‐regulation that often reflects external motivation (Ryan and Deci 2017). When nurses embrace self‐punishment, they might focus on seeking approval, meeting expectations, or avoiding failure, rather than using independent professional judgement or following their own internal values (Dunaetz et al. 2024). Encouragingly, our results suggest that awareness of one's own beliefs and assumptions and intrinsic goal setting could enhance autonomy and empower nurses to make more independent, value‐driven decisions in their practice. Self‐punishment was also negatively related to job satisfaction. This outcome may be linked to increased emotional distress, as self‐punishment often involves self‐criticism, guilt, and shame (Neck et al. 2019). Leadership at all levels plays a critical role in making nurses feel valued and satisfied. By fostering self‐compassion and promoting a culture of constructive feedback, nurse leaders can support nurses' growth, help them build a stronger sense of professional confidence, and reduce negative self‐perceptions, thereby increasing job satisfaction (Wong 2024).

Surprisingly, in our findings, self‐reward reduced professional autonomy; nevertheless, the observed negative statistical association was weak. This can be explained by external motivation aspects when nurses' practices are driven only by rewards from their organisations (Seitovirta et al. 2018). Rewards are, hence, perceived as incentives that are defined and provided from the outside. Further, self‐reward practices may be perceived as artificial because most Finnish nurses do not typically use them. Although this remains speculative, the finding that self‐rewarding was rated as the weakest self‐leadership practice in this study may provide some support for our own reflections. Further research on self‐rewarding is needed to enhance understanding and support its effective implementation in practice.

An expected finding of this study was that nurses who used more natural reward strategies experienced greater job satisfaction. This aligns with previous research findings (Kagan et al. 2021; van Dorssen‐Boog et al. 2022). Natural reward strategies mean concretely that nurses can influence working conditions and find aspects that support and satisfy their work. A favourable nursing practice environment, including smooth teamwork and good nurse‐physician relations, is important to nurses (Kagan et al. 2021). In addition, nursing management, staff and resources, professional autonomy, and quality standards in an organization contribute to the state of nurses' job satisfaction (Pursio, Kankkunen, Mikkonen, and Kvist 2024). While full influence cannot always be attained, it remains important to direct energy towards things that lie within one's sphere of influence (Neck et al. 2019).

In this study, nurses who experienced greater professional autonomy and better job satisfaction were less likely to consider leaving their profession. This finding was also expected, and it supports previous evidence (Wei et al. 2023). Nurses' sense of autonomy, which is built through experiences such as playing an active role in decision making, increases their job engagement (Junttila et al. 2023). On the other hand, conflicts in leadership, roles, goals, ethics, and values are harmful to nurses' job engagement (Aungsuroch et al. 2024). Organisations that give nurses the freedom to carry out their work individually and enable them to develop their professional role by providing the training and resources necessary suffer less from nurses' turnover intentions (Chu et al. 2021).

Nurses who evaluated their own beliefs and assumptions considered leaving their profession more often, while other self‐leadership practices were not associated with the intention to leave. In other words, in this study, we could not demonstrate that stronger self‐leadership reduced the intention to leave. This finding is unexpected in several respects. It is noteworthy and concerning that reflection on personal beliefs and assumptions may contribute to increased intentions to leave nursing. Evaluating personal beliefs and assumptions is part of constructivist thinking, which aims to identify dysfunctional and sometimes even unrealistic thought patterns (Neck et al. 2019). Reshaping one's way of thinking is challenging, particularly without intentional self‐reflection. For nurses, engaging in critical self‐reflection and active peer discussions enhances their awareness and ability to recognise ethical value challenges, conflicts, and the associated stress—potentially leading them to question their role within the profession (Eklund et al. 2025). This remark strongly underscores the importance of promoting self‐leadership training within the healthcare sector.

The lack of other observed associations between self‐leadership practices and intentions to leave in this study is somewhat inconsistent with previous research. For example, Kim and Kim (2019) found that nurses' self‐leadership was related to job embeddedness and their willingness to stay in their jobs. However, their study also indicated that all aspects of self‐leadership included in the model were indirectly related to the intention to leave, so their importance cannot be ruled out. Self‐leadership can increase efficiency and job satisfaction (Knotts et al. 2021), and its importance is emphasised for coping with work issues. Self‐leadership practices help individuals put work‐related concerns aside when not on duty, which is especially important for nurses because patients may be on their minds even in their free time (Al Mamari and Groves 2023).

### 6.1 Strength and Limitations

One strength of this study is that the data were collected using the RSLQ, which is an internationally validated and widely used instrument that covers self‐leadership strategies comprehensively (Knotts et al. 2021). However, this is the first study in the Finnish nursing context with a relatively small sample (*n* = 230). Still, the sample size exceeded a recommendation based on power analysis (*n* = 154). The low number of respondents may have been influenced by the research topic, which has not yet received much attention in nursing. It is important to consider, when interpreting the findings, that data obtained via self‐assessment measures may be subject to self‐report bias. Additionally, the data were collected from one organization, so the results can be considered only indicative and cannot be generalised to other organisations.

Another strength of the study is the examination of the relationships between self‐leadership, professional autonomy, job satisfaction, and intention to leave the profession using structural equation modelling. Hence, this study produced new information about self‐leadership and related factors in nursing, which were previously partly unknown. A limitation is that re‐coding the intention to leave as a dichotomous variable may obscure the complexity of the phenomenon under study and potentially lead to a loss of some information. Moreover, the use of regression imputation may underestimate standard errors and slightly overestimate model fit, as it does not account for the uncertainty associated with imputed values. The cross‐sectional nature of the study precludes any inference of causality.

### 6.2 Recommendations for Further Research

Overall, international research on nurses' self‐leadership is still limited. We need cross‐sectional studies along with comparative studies between countries to describe the research phenomenon and the factors associated with it to understand the complexity of the topic. More qualitative information should be collected about nurses' experiences and views on self‐leadership and professional autonomy in different nursing environments, which are currently also overshadowed by a high workload, a shortage of nurses, and various crises. Intervention studies are needed to demonstrate how self‐leadership skills can be developed. In the future, it is also important to further investigate how nurse leaders experience nurses' self‐leadership and what role self‐directed nurses play under their supervision.

### 6.3 Implications for Policy and Practice

Promoting nurses' autonomous motivation through organisational structures could be a crucial step in sustaining the nursing workforce (Gagnon et al. 2024). Therefore, both nurses' professional autonomy and self‐leadership practices should be considered for all their potential. Opportunities to participate in decision making and development, as well as to work in meaningful and enjoyable ways, help implement autonomy and natural reward strategies as part of an organization's culture and structures. Empowering leadership supports and encourages nurses to set self‐goals, evaluate beliefs and assumptions, and reward themselves for even small accomplishments. Moreover, high work pressure, competition, and emphasising mistakes maintain nurses' tendency towards self‐punishment, which can be avoided with an open and healthy work environment. Self‐leadership skills should be strengthened in nursing education, so that nurses are increasingly equipped for professional autonomy, job satisfaction, and retention.

## 7 Conclusion

Nurses in our study rated their self‐leadership practices as moderately good, and our findings suggest that setting personal goals and evaluating beliefs and assumptions are positively associated with professional autonomy, which in turn is linked to higher job satisfaction and lower intention to leave the profession. Goal setting and autonomy are intrinsic motivational pursuits that are beneficial for nurses' independent actions. By the same token, using constructive mindsets, such as identifying dysfunctional beliefs, develops ways of thinking that positively impact nurses' competence and readiness to perform autonomously. Natural reward strategies that involve performing tasks that a person finds meaningful, interesting, and challenging, while surrounded by empowering colleagues and superiors, increase job satisfaction. However, not all self‐leadership strategies are beneficial—self‐punishment can lead to lower professional autonomy and job satisfaction. In addition, evaluating one's own beliefs and assumptions, although linked to greater autonomy, might increase the intention to leave the profession due to reflective thoughts about enjoying (or not enjoying) the profession.

## Author Contributions

K.P.: Conceptualization and design, methodology, data collection, software, data analysis, tables and figures, writing – original and revised draft. A.N.: Conceptualization and design, methodology, data analysis, writing – original and revised draft. S.M.: Methodology, software, data analysis, writing – original and revised draft. T.K.: Conceptualization and design, methodology, data analysis, writing – original and revised draft. All authors: Final approval and agreement to be accountable for all aspects of the work.

## Disclosure

There is a statistician on the author team, Santtu Mikkonen, PhD, Docent.

## Ethics Statement

Ethical approval was obtained from the Committee on Research Ethics at the University of Eastern Finland on September 12th, 2024 (Statement No 17/2024), while Wellbeing Services County provided study permission on October 20th, 2024 (Hyvaks 20.102024). Permission for the use of the instruments was obtained from their copyright holders via e‐mail (Mrs. Dempster‐Gonzalez Oct 5th, 204 and Prof. Houghton Aug 31st, 2023).

## Consent

The authors have nothing to report.

## Conflicts of Interest

The authors declare no conflicts of interest.

## Supporting information


**Table S1:** Regression weights, unstandardized (Est.) and standardised (S.Est.).

**Table S2:** STROBE Statement—Checklist of items that should be included in reports of cross‐sectional studies.

## Data Availability

The data is not publicly available due to privacy or ethical restrictions. The data sets used and analyzed are available from the corresponding author on reasonable request.
