# Supplementary material for: Relationships Between Nurses' Self‐Leadership Practices, Professional Autonomy, Job Satisfaction and Intention to Leave: A Structural Equation Modelling Approach
Source: J Adv Nurs. 2025 Oct 23;82(6):6264–73. doi: 10.1111/jan.70316 (PMC13176705; doi:10.1111/jan.70316)
Supplement: Supplementary file 1 — Table S1: Regression weights, unstandardized (Est.) and standardised (S.Est.). [file JAN-82-6264-s001.docx]

Supplementary file 1.

Table S1. Regression weights, unstandardized (Est.) and standardized (S.Est.).

| Variables | | | Est. | S. Est | S.E. | C.R. | p |
| --- | --- | --- | --- | --- | --- | --- | --- |
| Job satisfaction | 🡨 | Natural reward strategies | 0.439 | 0.195 | 0.144 | 3.052 | 0.002 |
| Job satisfaction | 🡨 | Self-punishment | -0.300 | -0.175 | 0.109 | -2.748 | 0.006 |
| Professional autonomy | 🡨 | Job satisfaction | 0.102 | 0.390 | 0.014 | 7.193 | <0.001 |
| Professional autonomy | 🡨 | Self-goal setting | 0.161 | 0.244 | 0.048 | 3.322 | <0.001 |
| Professional autonomy | 🡨 | Self-reward | -0.059 | -0.147 | 0.024 | -2.480 | 0.013 |
| Professional autonomy | 🡨 | Evaluating beliefs and assumptions | 0.149 | 0.233 | 0.044 | 3.361 | <0.001 |
| Professional autonomy | 🡨 | Self-punishment | -0.115 | -0.255 | 0.026 | -4.420 | <0.001 |
| Intention to leave | 🡨 | Job satisfaction | 0.116 | 0.417 | 0.017 | 6.738 | <0.001 |
| Intention to leave | 🡨 | Evaluating beliefs and assumptions | -0.114 | -0.168 | 0.040 | -2.891 | 0.004 |
| Intention to leave | 🡨 | Professional autonomy | 0.240 | 0.226 | 0.069 | 3.482 | <0.001 |

Note: Est. = regression weight; S. Est = Standardized regression weight; S.E. = standard error; C.R. = critical ratio; p = probability level.
